# Ten quick tips for making things findable

**DOI:** 10.1371/journal.pcbi.1008469

**Published:** 2020-12-31

**Authors:** Sarah Lin, Ibraheem Ali, Greg Wilson

**Affiliations:** 1 RStudio, PBC, Boston, Massachusetts, United States of America; 2 Louise M. Darling Biomedical Library, University of California, Los Angeles, California, United States of America; University of Toronto, CANADA

## Abstract

The distribution of scholarly content today happens in the context of an immense deluge of information found on the internet. As a result, researchers face serious challenges when archiving and finding information that relates to their work. Library science principles provide a framework for navigating information ecosystems in order to help researchers improve findability of their professional output. Here, we describe the information ecosystem which consists of users, context, and content, all 3 of which must be addressed to make information findable and usable. We provide a set of tips that can help researchers evaluate who their users are, how to archive their research outputs to encourage findability, and how to leverage structural elements of software to make it easier to find information within and beyond their publications. As scholars evaluate their research communication strategies, they can use these steps to improve how their research is discovered and reused.

## Introduction

Researchers have always had to manage information, but the exponential growth of electronic data has both required and fostered the creation of new ways to do this [1,2]. The problem is not just about finding a particular article or website, but also how to find the information within that is important for reusability and interpretation of the work. Information may be stored in many formats, exist in multiple versions, and need to be shared with varied audiences for both research and teaching.

In the last 4 years, the growing adoption of findable, accessible, interoperable, and reusable (FAIR) principles [3] has helped researchers manage the data and other digital objects associated with their professional work. Indeed, research reproducibility hinges on these 4 principles, which form the backbone the following 10 tips. This paper is concerned with findability, including research data and extending beyond it to encompass all types of work products produced during a researcher’s career, inside or outside academic settings.

Library science offers ways to work through the maze of information generated in professional life, and librarians’ skills can be applied by any researcher who seeks to improve access to—and utilization of—their research and other professional outputs. The 10 quick tips in this paper build on the fact that all information ecosystems have users, context, and content [1]. To solve the information retrieval problem, researchers must therefore think broadly about who needs that information and the context within which it is created, as well as its actual content.

### Design for a wide range of users

The first step in making information findable is to determine who will be doing the finding. This includes everyone who might learn from your work, contribute to it, expand upon it, or re-share information through their own networks [4]. While you might think that there are only a few relevant experts who know your field well, novices and trainees will also need to use your work as they gain experience in the field, thereby making your actual user base considerably larger and more diverse. Furthermore, some users need to access scholarship through an intermediary, such as translation software or a screen reader. Being mindful of all potential users and how they might need to interact with you and your work is the foundation of all 10 tips.

The information you wish to convey and the way it is currently organized may make perfect sense to you, but its meaning for your users is determined by what they interpret from the information they encounter and the way it’s arranged. This means that the organizational strategies you use are a communication channel in their own right. To illustrate this, the author Jorge Luis Borges created a classification of animals whose categories included “those belonging to the Emperor,” “embalmed ones,” “suckling pigs,” “those included in this classification,” “those drawn with a very fine camel hair brush,” and “those that look like flies from far away” [5]. While this was deliberately ridiculous, it illustrates the fact that every way of organizing knowledge embodies choices by the organizer, which may or may not align with those of the audience. Therefore, when preparing materials for sharing, it is important to establish context, to use clear and concise language, and to minimize the use of jargon.

More concretely, consider the website of a faculty member coming up for tenure: She created the website as a postdoc to publicize her papers and to make it easier to fill out grant applications by listing professional activities in 1 place. Yet the site might be useful in other cases as well:

- Colleagues may come to the site looking for un-paywalled copies of her papers, to find out what she’s currently working on, or where she is next going to present her work.
- Tenure committee members might review her accomplishments to assess her work’s impact.
- A librarian (or a program written by a librarian) might scrape that site for journal articles to include in the university’s institutional repository.
- A student might come to the site looking for course information or materials.

Reaching out to a variety of users with distinct needs to ask for findability feedback will help you discover any gaps in organizational alignment.

### Design with the end in mind

Given the current state of technology, it can be easy to rapidly create digital information. With the plethora of software and formats you may employ, you almost certainly have information in lots of different formats, file types, and locations. Furthermore, the surge in the use of preprints and other similar research products has created an abundance of citable research products. While they are useful for demonstrating open research productivity, they create an added challenge of finding the many different types of research products that arise in the modern workflow. The second step in making things findable is to think ahead about the things you would want to be found at the completion of the project, including relevant preprints, before cataloging what you have. That way, you can have time to anticipate what can go where and adapt if necessary before materials are published.

**Fig 1:**
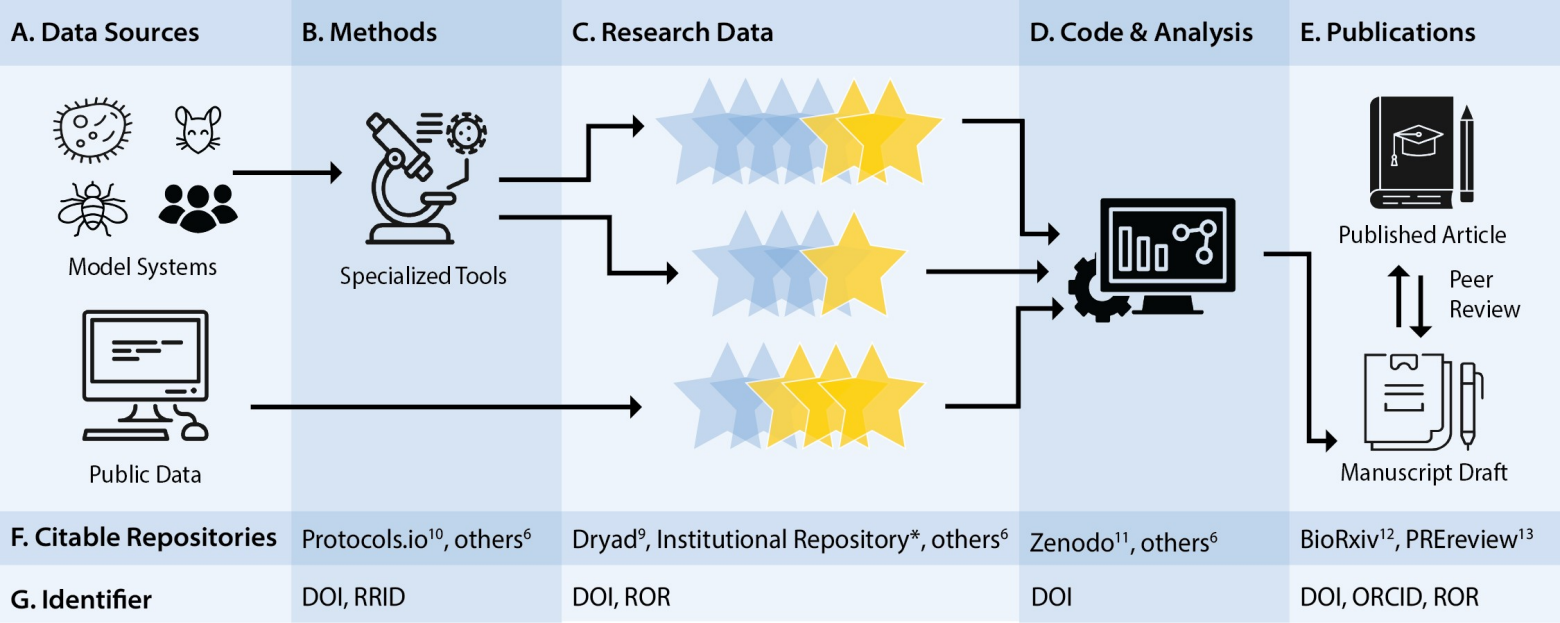
A typical research workflow. (A–E) Major steps of the research workflow. (F) An example set of citable repositories or tools used to find research products outside of journal articles. (G) Persistent identifiers used for the example repositories listed. DOI, digital object identifier; ROR, Research Organization Registry; RRID, research resource identifier; ORCID, open researcher and contributor ID.

This is particularly relevant when examining a typical research workflow. A researcher uses a particular set of data sources (Fig 1A), such as model organisms, molecular systems, publicly available next-generation sequencing data, or other locally curated collections of information. Subsequently, data sources are treated with specialized protocols and tools in the lab (Fig 1B) which help visualize, identify, or extrapolate new observations about biological phenomena (Fig 1C). After repetition, trends seen among noisy biological observations can be further analyzed, visualized, and statistically evaluated with software or code (Fig 1D). Finally, the researcher builds context for the work by writing a manuscript and citing relevant literature (Fig 1E). Manuscripts are revised by colleagues in the field through peer review and published in a peer-reviewed journal.

By planning ahead, you can identify what research products can go where and update them if necessary before materials are published in a peer-reviewed journal or cited in a grant application. Advancements in best practices for making research products more “FAIR” has led to the creation of an array of discipline-specific and general repositories [6] (Fig 1F). Researchers can archive and find research products created from each of the steps in the workflow. Many repositories now create digital object identifiers (DOIs) [7] or other permanent identifiers for their submissions, making them citable and easily linkable to the contributing researchers via open researcher and contributor IDs (ORCIDs) [8] (Fig 1G). Many repositories follow the standards set by experts in the field, reporting information that is recommended for reproducibility while employing necessary practices to protect the privacy of sensitive information.

Elaborating on our example of the hypothetical faculty member, she wants to plan ahead to be sure her research is accessible and reusable by her lab, students, and collaborators. She works in a competitive field, so she also wants to maintain some privacy with regard to works in progress. She takes advantage of some repositories and tools that help make her work easier to find for a variety of users (Fig 1F). This way nearly all of her major research products can be citable and are interlinked. The resulting network of citations aids findability greatly:

- Finalized laboratory data is stored in Dryad [9], which allows her to cite the same data source in multiple publications with a single DOI. She can even update the data archive as new pertinent data is collected and the link will not change.
- Lab protocols maintained in Protocols.io [10] ensure that future collaborators, lab members, and students can easily find her established protocols using a DOI, while keeping incomplete protocol drafts private.
- Code used to analyze the data is deposited in Zenodo [11], which she can cite and update as code gets optimized and new versions are created, which also allows anyone looking to reproduce her research to also reproduce the analysis.
- Posting finalized drafts of her student’s research manuscripts on a preprint site like BioRxiv [12] enables colleagues to download the draft easily, discuss in an upcoming journal club, and publish the feedback publicly in PREreview [13].
- Her ORCID identifier is linked to each of the DOIs created by these repositories, ensuring that students, collaborators, tenure committees, and librarians can all access her research outputs with 1 link.

Following the standards recommended by experts [3] in the field as you design, your information ecosystem will reduce the barrier to finding relevant materials associated with your work. Furthermore, most publishers already require data and code archiving to encourage reproducibility. Remember that your future self is also 1 of your users: Everyone is prone to forgetfulness, so anything you do for others will likely pay off for yourself eventually [14].

Planning to incorporate persistent identifiers into the research workflow is a straightforward way to design for findability. Remember too that the strategies users employ to find something can look different in different contexts. Beyond finding your published or unpublished work on the web, users may need to find a specific item within a website and/or find a particular piece of information within a specific file or item within a webpage. Depending on your content, you may have challenges in all 3 areas; the remaining tips on context and content will help you address these issues.

### Use textual structure

Findability at the document, post, or article level can be improved by taking advantage of the textual structures that information management programs provide [2]. For example, a key part of searching the web is scanning the text returned by search engines to see if it contains target information. Textual structure helps that process [15]: Formatted headers (rather than just enlarged text), bulleted or numbered lists, and **highlighting** terms that are important all make both the information and its structure easier to understand. Similarly, headings and table of contents can be hyperlinked, which supports both scanning and navigation. Textual structure aids navigation by not only helping users create a mental map of the webpage or document they have found, but also by exposing elements utilized by screen readers to make your work accessible.

Textual content is created and aggregated in so many forms, using so many different programs, that it is difficult to specify strategies beyond headings, lists, and highlighting. However, specialists working in the same field tend to adopt the same tools, so it is worth exploring how your peers annotate information as well as creating, manipulating, or storing it. For example,

- GitHub allows users to add tags to issues and commit messages which can then be searched across projects.
- Electronic lab notebooks can use XML schemas like Darwin Core, electronic mail format (EML), or flexible image transport system (FITS) [14].
- Using specific Google Docs heading levels creates a table of contents in real time, visible when the file is open.
- Comma-separated values (CSV) files do not have a standard way to store metadata, but authors commonly created a README or MANIFEST file that describes the structure and content of the files in a collection. (See [16] for examples.)

On a practical level, templates for file creation, data collection, and electronic lab notebooks make it easier to be consistent and to spot inconsistencies.

### Add metadata

Just like people who end up with piles of photographs with nothing written on the back, we all have digital mounds of files and content with no metadata describing when it was created or what it contains. Even the most basic metadata provides extra clues for information retrieval; however, what you can add depends on the software you use to create, store, and access your information, and on the file formats that information is stored in.

Almost all modern operating systems allow you to add information to the properties of a file or directory. Databases, word processors, and website construction programs also have built-in metadata capabilities, although they may be hard to find and harder to understand how to leverage. To make matters worse, the fact that metadata is often software specific makes it easy for inconsistencies to creep in. For example,

- The tags used on a WordPress website may not be in step with the properties in the images on that site.
- When a citation is copied from a database or repository to a bibliography manager, the software may not copy over the structural information implied by the article’s location in the database.

The most difficult thing about metadata, however, is getting into the habit of creating it in the first place. If you get to choose what software to use, it helps to pick one that simplifies metadata creation. For example, most website generators allow you to type tags into an article’s header without having to define them first. This can lead to a proliferation of synonymous (or misspelled) tags, but some occasional cleanup is better than tackling a mountain of untagged information. Repositories that force metadata creation upon submission greatly assist efforts to make work product findable, and researchers would be well served to replicate those metadata elements within their own file storage schemas. Indeed, creating an internal taxonomy (list of terms) or ontology (list of relationships) at the beginning of a project can make assigning metadata much easier.

You should also examine how metadata can be transferred from an old system to a new one if you have the luxury of switching software (or have a change forced on you). Some form of XML is usually the best option when doing this: It is likely to be with us for many years to come, and the same pedantry that makes it tedious for human beings to type and read ensures that programs can read it without having to guess what its creators actually intended. FAIR principles help ease the burden of software and storage migration. They encourage researchers to plan for interoperability in software and data storage from the beginning of their projects and reduce concerns about data migration.

### Use search and browsing

Research on information seeking shows that people search and browse when they’re trying to find information. As they browse a website, document, or file, they build a mental map of the content they could possibly find and then search based on that map. “In the process, they modify their information requests as they learn more about what they need and what information is available from the system” [1]. You have probably seen or done something similar with a print book, trying to determine if it’s the one you want by looking at the table of contents and the back cover. These 2 functions work together because search allows users to find information they know they need, whereas browsing allows users to find information they don’t know that they need [17]. Designing for both browsing and searching is especially pertinent in a search algorithm environment that often dynamically creates results unique to each individual search executed, influenced by the user’s previous interaction with a particular website and/or internet browser.

You should therefore make information accessible both ways and make it easy to move from searching to browsing and back again. Tags and other metadata help with searching, while structural clues tell users about the content contained in the information they are looking at. That communication “enables the answers to users’ questions to rise to the surface and answer questions like, Where am I? What’s here? Where can I go from here?” [1]. Similarly, “… the words you use in the navigation systems and headings of [your content] help you find what you’re looking *for*, but they also help you understand what you’re looking *at*” [18].

For example, when users don’t know exactly what they need, the terms in a menu help them understand the vocabulary used in this domain and the boundaries of what is included (i.e., terms are listed) or excluded (i.e., no menu terms exist). At the same time, the headings in the documents they find act as topical markers: They help users not only summarize the information contained in the document, but also refine what they would search for based on the terms used in those headings. Navigation bars on websites function in a similar way: If the user knows exactly what they are looking for, they can scan the menu and select the option that matches their need.

Additionally, users might want content organized chronologically or topically. In websites that are generated programmatically using tools such as Blogdown [19] or Wordpress [20], these types of organizational features may be simple to implement. However, increasing the number of site navigation options may confuse the user if it is unclear how items relate to each other. Authors must strike a balance between offering filtering and searching options and being as inclusive as possible when deciding how materials will be structured and shared with their users.

### Mimic real-world directions

The language we use in digital environments mirrors that used for physical directions: We “visit” or “go to” a website without actually changing our physical location. Using the navigational metaphor consistently helps users build the mental map mentioned in the previous tip. File paths and breadcrumb trails on websites give users a sense of where the information resides and suggest new paths they can take [15]. For example, the uniform resource locator (URL) of a website might all include the name of a section of the site, such as /papers/ or /blog/. DOIs and ORCIDs function as the best kind of internet “signage,” ensuring that users never encounter a “webpage not found” error message in the course of retrieving a particular publication.

While much has been written about web usability in general [4,21], library science focuses on information-seeking behavior. For example, we know that users scan but don’t read: They click on the first close thing they see and give up very, very quickly [17]. Your markers and directions should therefore be as consistent as highway signs with regards to appearance, style, and type of information. Wherever possible (and it’s always possible), use mechanisms that users will have become familiar with elsewhere, such as the vertically nested folders of file browsers or the left-to-right arrangement of breadcrumb trails. Persistent identifiers are a great example of markers that consistently aid navigation in structured (e.g., databases) and unstructured (e.g., personal websites) environments.

### Use meaningful names

The names of files and URLs of webpages are the 1 piece of metadata you cannot avoid creating, so always choose ones that are human-readable and that convey information about what they name, both when navigated to and when returned in search results. Returning again to the faculty member’s website, it would be easy to name a paper plos2020.pdf, but since other people may also have published papers in PLOS in 2020, a more structured name such as lin-findability-plos-2020.pdf will both convey more information at a glance and retain that information after the paper has been downloaded and put in a folder with dozens of others.

There are many ways to develop a naming schema, largely related to the nature of the information you create. At the most basic level, you should use consistent names for the same reason that you use good file organization, so you can easily find and use data later. Additionally, good naming helps you avoid duplicating information [14]. Researchers with multiple research projects or significant complexity in their data sources should establish and document a unified system of abbreviations for those projects or sources; these can be summarized in a data dictionary or README file. Consistency is key: Standardizing on lower case, a preferred date format (YYYYMMDD or YYYY-MM-DD will both sort chronologically), and filename suffixes (.jpg instead of .jpeg) will help everyone find what they need [22,23].

Renaming existing files to be consistent with your standards after the fact can seem like a waste of precious time, but since the research cycle doesn’t end with publication [14], there is a very high likelihood that someone will need to reuse your data and will have to try to figure out what files corresponded to what part of your research. Similarly to establishing metadata norms before beginning a project, creating a naming convention that is adopted by any collaborators before research begins will preserve findability into the future.

If you have things to name that are not files, such as projects, web pages, or document headings, remember that the more generic a term is, the harder it is to search for: Naming a raw data file “raw” or a downloaded file “download” makes finding the information they contain nearly impossible. A quick test is to search for the name before adopting it: If dozens of unrelated results come up, you may want to pick a different name. You should also think about nicknames or shortened versions of your names and make sure they are present in text or tags so that the content can be discovered by a search engine and a user.

### Use tags

After meaningful names, tags are the easiest and most effective metadata you can create. Almost all digital tools allow users to add arbitrary tags to items: File properties on Windows and labels on GitHub issues are just 2 examples. Additionally, almost all search tools leverage tags to narrow a query’s scope. This means that you can now file a single thing in multiple “locations,” which was not possible in the pre-digital era. Multiple tags also assist users from varied backgrounds because the terms can be customized to be inclusive of a diverse set of users.

When choosing tags, be consistent in your depth of topical term assignment (how specific your terms are) and your selection of terms for subject and format (the number of terms you use to describe each subject and format). For example, if you tag some items in an ecological data set with a species name, don’t tag others simply as “reptile” unless the species is unknown, in which case you should

- Tag all items “reptile,” “bird,” “mammal,” and so on for high-level searches, and
- Tag all items with a species, which might be “unknown” or “NA” (not available), or
- Tag all items at both general and increasingly specific categories if that is the standard for your discipline [24].

What should you tag? The answer is “everything” from informal personal notes to data sets submitted with publications or included in repositories, because it is all the material you will want to be able to find later. The benefit of tagging comes from doing it in all of those situations, not just when a journal submission requires it.

If you are certain something is for purely personal use, you can create your own taxonomy of subject keywords, which is called a folksonomy. Folksonomies are what you see with tags on Flickr: Early content creators assign terms as they see fit, and later contributors can use those or add their own. If you take this route, it’s worth reviewing new tags regularly to look for synonyms, misspellings, differences in capitalization, singular/plural discrepancies, and other inconsistencies.

What terms you use as tags for personal consumption may not matter much, but work that is shared with colleagues should use particular terms or tags that conform to relevant standards [24]. These terms typically come from taxonomies, thesauri, and ontologies: Taxonomies and thesauri generally have built-in subject hierarchies that can help you create navigational structure, while ontologies map relationships between ideas. Crucially, all 3 are controlled vocabularies: They are a defined list of terms created and maintained by experts rather than being crowdsourced like a folksonomy.

There may or may not be relationships built between terms in a controlled vocabulary, such as equivalencies (“CA” for “California”), broader/narrower terms (United States/California), and/or replacement (Weed, Use Marijuana). Established subject terms will match article databases, data repositories, and library catalogs that you and your users might already be familiar with, which will again aid search and navigation. Well-known examples in the United States include the National Cancer Institute (NCI) Thesaurus [25] and the Medical Subject Headings (MeSH) [26].

### Understand the difference between format and subject

However you create tags, you need to address the distinction between format and subject. Format describes what your content “is,” while subject describes what it is “about” [27]. About-ness is the most common content analysis, but isness issues will probably affect people’s ability to use your information, so you may want to add metadata to make it explicit.

A simple example of this is a blog post on a researcher’s professional website. The post is “about” a subject, like a book review, but it “is” a blog post rather than biographical details, bibliography, or a list of currently taught classes. Going back to your users, what subjects are important to them? And do those topics carry over or change between differences in format? For a librarian, this is basically a question of combined terms: Are your format terms uniquely matched to topics (e.g., blog posts are always about news), or do you have multiple topics in each format (e.g., blog posts and tutorials on the same subject)?

Similarly, you can rely on filename suffixes to distinguish computational notebooks from PDF files, tabular data sets, or slide decks, but should use tagging, a filename convention, or a description in a README to tell people whether the contents are raw information, tidied-up data, or an aggregation of several underlying data sets. This enables users to search by topic, format, or both.

Since dissemination sometimes changes a file’s format (e.g., printing slides to a PDF), naming and metadata conventions tend to be more robust as well as more informative than relying on file types. Once again, structural clues can help: A folder specifically for conference presentations may contain 1 subfolder for each presentation, which, in turn, contains the PowerPoint and PDF versions of the presentation with exactly the same names but different filetype suffixes. Likewise, journal articles you store will need a naming or structural convention to distinguish articles you have written from those you have downloaded for your own use.

### Do not abbrvt

Acronyms and abbreviations make communication between those who know them more efficient at the price of making them less accessible to newcomers. Spelling out acronyms and abbreviations that you take for granted (or hyperlinking to their definitions) makes information easier to find and enables newcomers to participate in conversations that are considered technical or advanced. When doing this, remember that acronyms are often repurposed by different professions or disciplines: What seems obvious to you is probably not obvious to people from other communities. Since every discipline has some common abbreviations, write them all out in full the first time they appear or create or point to a term dictionary.

## Conclusions

Changing work habits is hard, so remember that while perfection isn’t possible, progress is. Start by deciding whether to begin your next project with a new set of information organizing principles or to go back and alter existing artifacts [14]. You might also consider this process as you would a research experiment and incorporate 1 small change at a time. Whichever you choose, the “ways you enforce your way of doing things changes how users think about the place[s] you made and perhaps ultimately, how they think about you” [4].
